# *In vivo* and *in vitro* sex differences in the dendritic morphology of developing murine hippocampal and cortical neurons

**DOI:** 10.1038/s41598-017-08459-z

**Published:** 2017-08-16

**Authors:** Kimberly P. Keil, Sunjay Sethi, Machelle D. Wilson, Hao Chen, Pamela J. Lein

**Affiliations:** 1 Department of Molecular Biosciences, School of Veterinary Medicine, University of California Davis, Davis, CA USA; 2 Clinical and Translational Science Center, Department of Public Health Sciences, Division of Biostatistics, University of California, Davis, CA United States

## Abstract

Altered dendritic morphology is common in neurodevelopmental disorders (NDDs), many of which show sex biases in prevalence, onset and/or severity. However, whether dendritic morphology varies as a function of sex in juvenile mice or primary neuronal cell cultures is largely unknown even though both are widely used models for studying NDDs. To address this gap, we quantified dendritic morphology in CA1 pyramidal hippocampal and adjacent somatosensory pyramidal cortical neurons from male and female postnatal day (P)28 C57BL/6J mice. As determined by Sholl analysis of Golgi-stained brain sections, dendritic arbors of male hippocampal neurons are more complex than females. Conversely, dendritic morphology of female cortical neurons is more complex than males. In primary neuron-glia co-cultures from P0 mouse hippocampi, male neurons have more complex dendritic arbors than female neurons. Sex differences are less pronounced in cortical cultures. *In vitro* sex differences in dendritic morphology are driven in part by estrogen-dependent mechanisms, as evidenced by decreased dendritic complexity in male hippocampal neurons cultured in phenol red-free media or in the presence of an estrogen receptor antagonist. Evidence that sex influences dendritic morphogenesis in two models of neurodevelopment in a region-specific manner has significant mechanistic implications regarding sex biases in NDDs.

## Introduction

While neurodevelopmental disorders (NDDs) such as autism spectrum disorder (ASD), attention deficit and hyperactivity disorder (ADHD) and schizophrenia affect both sexes^1, 2^, they show a clear sex bias in onset, severity and/or prevalence^3, 4^. For example, ASD is almost five times more common among boys (1 in 42) than girls (1 in 189)^5^; ADHD symptoms differ in boys and girls with boys tending to show externalizing problems while girls exhibit internalizing problems^6–8^; and men tend to develop schizophrenia earlier and in a more severe form than women^9^. However, despite the importance of sex in NDDs, the mechanisms underlying sex differences are not completely understood. Addressing this gap is necessary for improving preventative, diagnostic and therapeutic strategies for NDDs.

Inherent sex differences in neurodevelopment may contribute to the sex bias observed in NDDs. The vertebrate brain is organized in a sex-dependent manner^10–13^, and permanent sex differences in the brain are established during development by gonadal hormones^14–17^. Quantitative sex differences observed shortly after birth include overall size and organization of brain regions, the number and architecture of cells, and neurochemistry^18–26^. In rodent models, sex differences are especially evident in regions of the brain important for reproduction and sexual behavior, but are also observed in regions involved in cognition, learning and memory, such as the hippocampus and cortex^24, 27^, which correlates with sex differences in cognitive function in adult rats^27, 28^. Sex differences in the hippocampus and cortex are of particular interest since these regions are implicated in the etiology of multiple NDDs^1, 29–31^. Sex differences in the brain are maintained at later developmental stages and into adulthood, as exemplified by human neuroimaging studies demonstrating that the hippocampus and cortex continue to develop in a sex-dependent manner during adolescence during which time the volume of the hippocampus continues to increase in males but decreases in females^12, 32–35^.

More recent data demonstrate sex differences not only in brain structure, but also in neuronal cytoarchitecture, specifically the morphology of dendrites. This is critically important because dendritic morphology is a major determinant of neuronal connectivity^36–38^, which is often perturbed in NDDs^8, 30, 31, 39, 40^. Further, genes important for regulating dendritic shape and size during development are implicated in NDD etiology^40^. Sex differences in dendritic morphology have been observed in the neurotypical adult brain. For example, in adult rats, the dendrites of cortical neurons are longer, more complex and have greater spine density in males compared to females^41, 42^. In the CA3 hippocampus, dendritic morphology is more complex in female *versus* male neurons in the proximal portion of the arbor, while the distal dendritic arbor is more complex in male *versus* female neurons^43^. However, detailed analysis of dendrite morphology between sexes is lacking for mouse models of juvenile development and for *in vitro* models of neurodevelopment, both of which are experimental models commonly used for investigating pathogenic mechanisms of and therapeutic strategies for NDDs^44^.

Our preliminary studies of Golgi-stained neurons in the hippocampus of P28 C57BL/6J mice suggested that the basilar dendritic arbor of CA1 pyramidal hippocampal neurons is significantly more complex in male compared to female hippocampal neurons^45^. Here, we extend those studies to further characterize sex differences in the dendritic morphology of CA1 pyramidal hippocampal neurons and determine whether sex differences are generalized to pyramidal neurons in the somatosensory cortex of P28 mice. To determine whether sex differences are also observed *in vitro*, we quantified dendritic arborization in primary cell cultures derived from the hippocampus and neocortex of male *versus* female mouse pups at P0, which are widely used models for characterizing factors that influence dendritic arborization^46, 47^.

## Results

### Sex-dependent differences in dendritic complexity vary between brain regions in P28 mice

For these studies, we used Golgi staining to visualize individual pyramidal neurons in the CA1 hippocampus and adjacent somatosensory cortex of P28 male and female juvenile mice, an age previously examined for effects of early life stresses, including environmental exposures, on neurodevelopment^43, 48, 49^. The dendritic morphology of Golgi-stained neurons in brain sections from P28 C57BL/6J mice was quantified using Sholl analysis, a method in which concentric rings are placed at fixed intervals from the soma and dendritic intersections counted at each ring. Sholl plots obtained from Golgi-stained neurons were analyzed using a mixed-effects model to account for the structure of the data, in which multiple neurons were analyzed per animal, as well as the autoregressive covariance structure of the Sholl profile^45^. Dendritic arborization was analyzed as the area under the curve (AUC) in Sholl plots.

**Figure 1:**
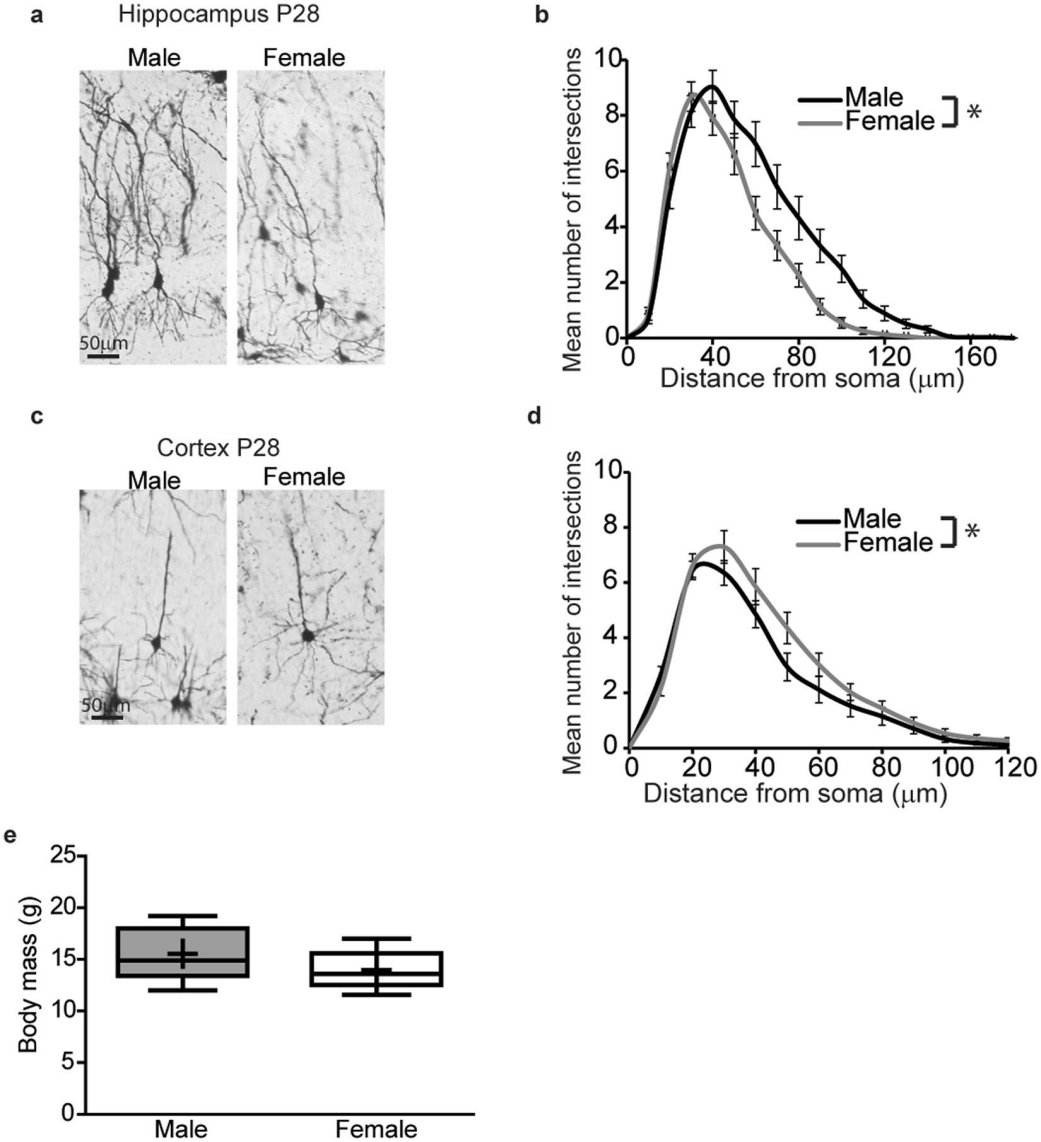
Dendritic arbors are more complex in male vs. female pyramidal neurons in the CA1 hippocampus while the opposite is true for pyramidal neurons in the adjacent somatosensory cortex *in vivo*. Representative photomicrographs of Golgi stained neurons (**a**,**c**) and Sholl analyses (**b**,**d**) of the basilar dendritic arbors in Golgi stained pyramidal CA1 hippocampal neurons (**a**,**b**) and adjacent pyramidal somatosensory cortical neurons (**c**,**d**) from postnatal day (P) 28 male and female C57BL/6 J mice. Body mass of P28 male and female mice (**e**). Data in panels b and d presented as mean ± SEM, (n = 35 male and 31 female hippocampal neurons, n = 34 male and 34 female cortical neurons from five animals of each sex from independent litters). In the box plots in panel e, “+” indicates the mean; whiskers, the 10–90^th^ percentile; n = 5 litter-independent mice per sex. Significant differences were determined using a mixed effects model for Sholl data (b, d) and Student’s T-test for body mass (**e**). Asterisk indicates a significant difference between groups at p ≤ 0.05.

Representative photomicrographs of Golgi-stained CA1 pyramidal hippocampal neurons and the corresponding Sholl plots are shown in Fig. 1(a and b). Based on Sholl analysis, male hippocampal neurons are significantly different from female hippocampal neurons (Fig. 1b, p < .0001). There is an increased number of intersections between dendrites and Sholl rings, particularly in the distal dendritic arbor, in males vs. females, indicating that the dendritic arbors of male hippocampal neurons are more complex than those of female neurons. There were no significant sex differences in the number of primary dendrites, defined as dendritic processes that originate at the soma (male 4.8 ± 0.4 vs. female 5.5 ± 0.4, p = 0.3), or in the area of the soma (male 243 ± 12 μm^2^ vs. female 270 ± 16 μm^2^, p = 0.3).

To determine whether sex differences in dendritic arborization are region specific, we also analyzed the dendritic morphology of pyramidal neurons in the somatosensory cortex of P28 mice (Fig. 1c). In the cortex, dendritic complexity is significantly increased in female *versus* male neurons (Fig. 1d, p = 0.0006); however, there are no significant differences in the number of primary dendrites (male 4.6 ± 0.2 vs. female 5.1 ± 0.2, p = 0.1) or in area of the soma (male 193 ± 11 μm^2^ vs. female 192 ± 11 μm^2^; using log10 transformation to improve conformation with model distributional assumptions, p = 0.96). Because experimental evidence indicates a positive correlation between body size and the extent of dendritic arborization in sympathetic neurons^50^, and since body mass has been correlated with neuron cell size in the brain^51^, we quantified body mass and found no significant differences between the sexes at the stage examined (Fig. 1e, p = 0.3).

### Sex differences in dendritic morphology in primary neuronal cell cultures

Dissociated cultures of primary neurons are a widely used experimental model for investigating mechanisms underlying normal and aberrant dendritic morphogenesis^46, 47^. However, the vast majority of published studies use cultures derived from tissues pooled from male and female pups within a litter to increase cell yield, especially when utilizing embryonic or early postnatal neurons^52, 53^. Therefore, in order to determine whether sex-dependent differences in dendritic morphology also exist *in vitro*, we set up primary neuron-glia co-cultures from male vs. female P0 mouse hippocampus or neocortex. On day *in vitro* (DIV) 6, high cell density cultures were transfected with a plasmid encoding microtubule-associated-protein-2B (MAP2B) fused to enhanced green fluorescent protein (EGFP) under conditions of low transfection efficiency in order to visualize the complete dendritic arbor of individual neurons^52^. Cultures were then fixed on DIV 9 and the dendritic arbors of EGFP-positive cells were quantified by Sholl analysis and morphometric analyses of dendrite number and length.

**Figure 2:**
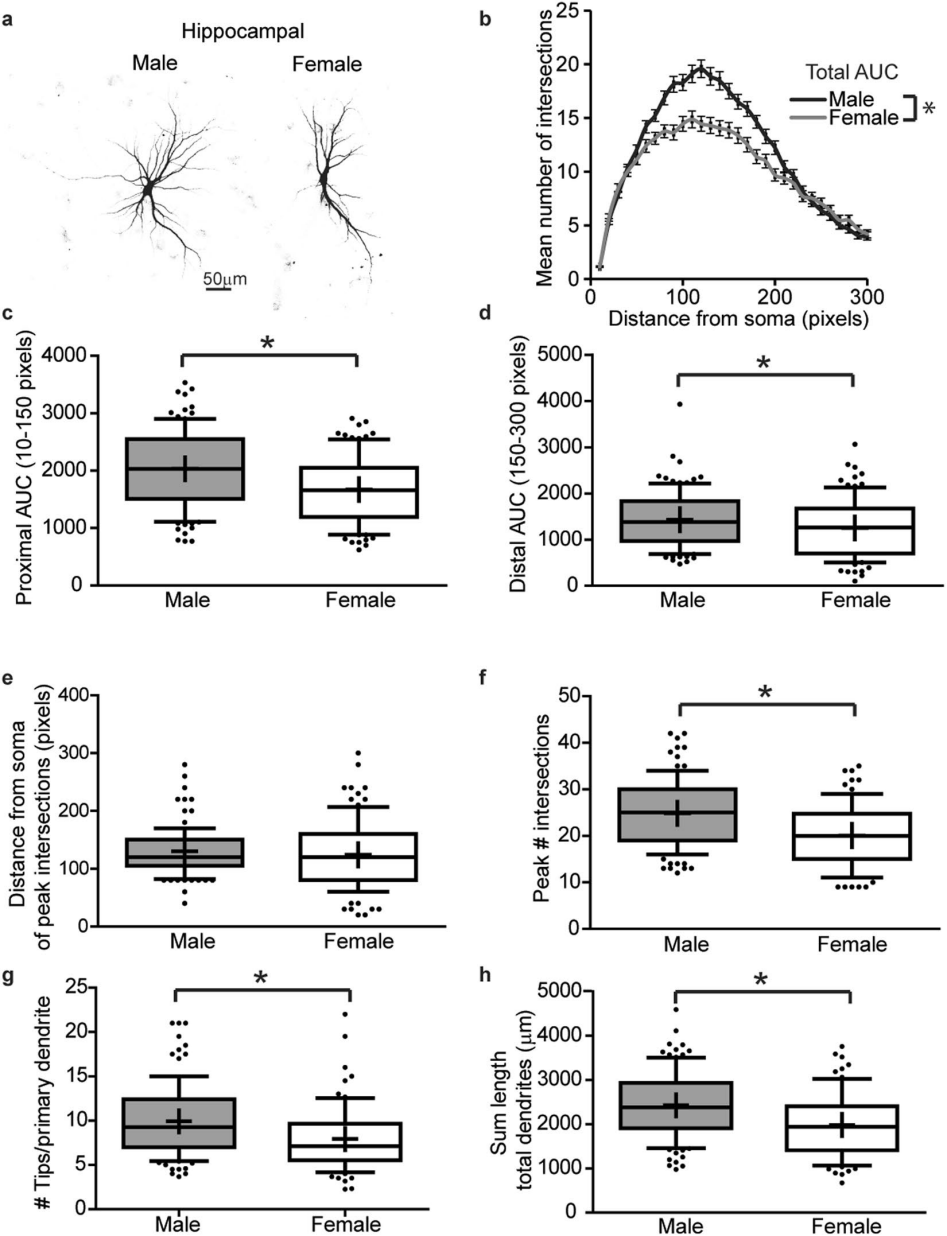
*In vitro*, dendritic arbors are more complex in male vs. female hippocampal neurons. Sex-specific neuron-glia co-cultures were established from P0 male and female mouse hippocampi, transfected with MAP2B-GFP plasmid on day *in vitro* (DIV) 6 and fixed on DIV 9. Representative images (**a**) and Sholl plot (**b**) of DIV 9 GFP-positive male and female hippocampal neurons. Dendritic morphology was assessed by quantifying: (**b**) the total area under the curve (AUC) in Sholl plots (10–300 pixels from the soma); (**c**) area under the proximal portion of the Sholl curve (10–150 pixels from the soma); (**d**) area under the distal portion of the Sholl curve (150–300 pixels from the soma); (**e**) distance from the soma at which the peak number of dendritic intersections occurs; (**f**) the peak number of dendritic intersections; (**g**) the number of terminal dendritic tips per primary dendrite; and (**h**) the total dendritic length per neuron. In the box plots (**c**–**h**), “+” indicates the mean; whiskers, the 10–90^th^ percentile, (n = 76–101 neurons per sex from at least five independent dissections). Significant differences were determined using Student’s T-test for parametric data (**b**,**c**,**f**,**h**) and Mann-Whitney U test for nonparametric data (**d**,**e**,**g**). Asterisk indicates a significant difference between groups at p ≤ 0.05. AUC = area under the curve. Magnification; 0.65 microns per pixel.

The dendritic morphology of primary hippocampal neurons varies between the sexes (Fig. 2). Specifically, dendritic complexity is significantly increased in male vs. female hippocampal neurons as assessed by the area under the Sholl curve for the total number of dendritic intersections at 10–300 pixels from the soma (Fig. 2b, p = 0.0005), as well as for intersections occurring in the proximal (10–150 pixels from the soma) (Fig. 2c, p = 0.0001) and distal (150–300 pixels from the soma) halves of the dendritic arbor (Fig. 2d, p = 0.04). Further, while the distance from the soma of the peak intersection did not differ significantly between sexes (Fig. 2e, p = 0.25), the maximum number of intersections is significantly greater in male compared to female hippocampal neurons (Fig. 2f, p = 0.0001). Additionally, the number of terminal dendritic tips per primary dendrite (Fig. 2g, p = 0.0003), and the total sum length of dendrites (Fig. 2h, p < 0.0001) are significantly greater in male compared to female hippocampal neurons.

**Figure 3:**
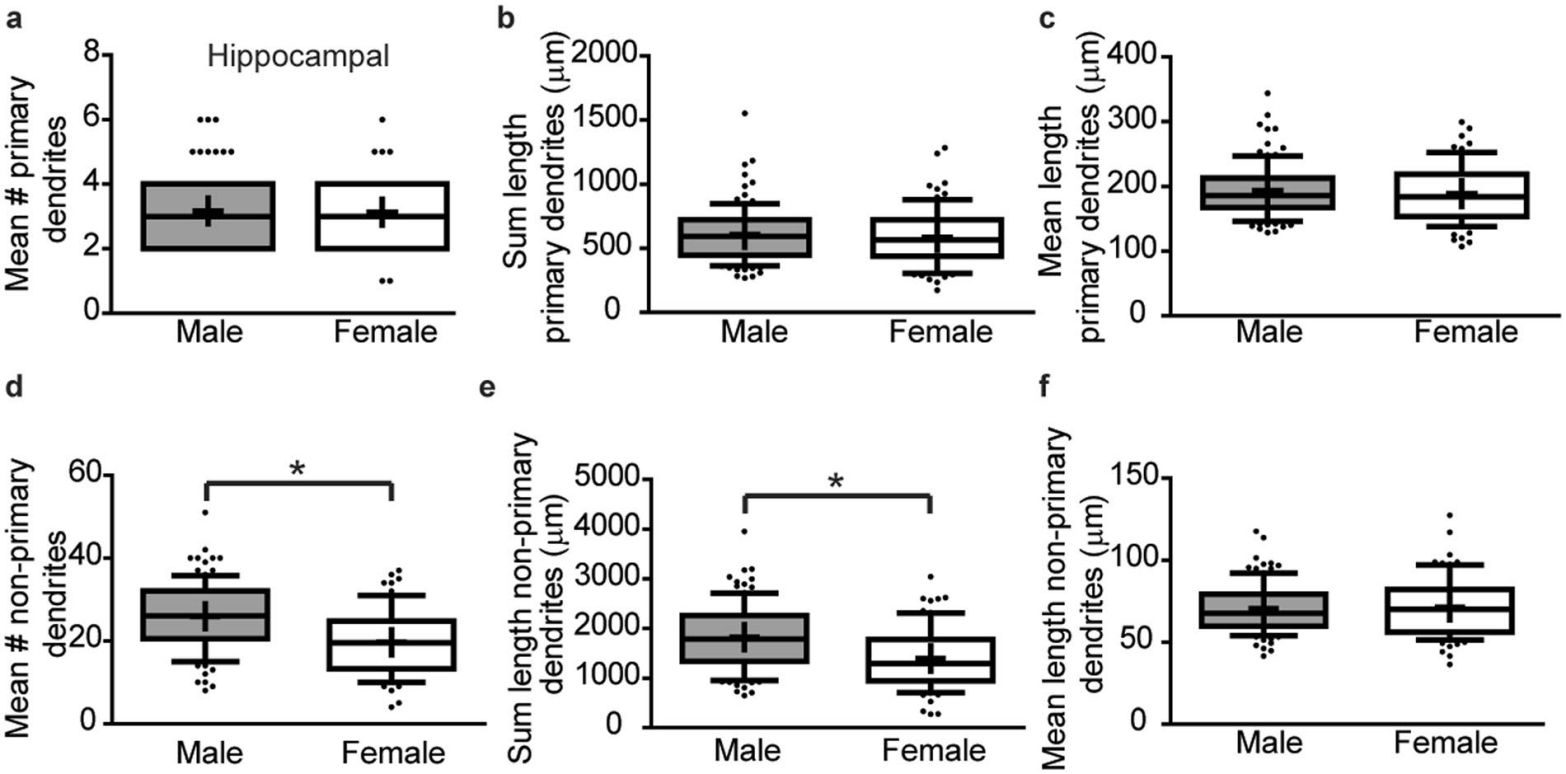
*In vitro* sex differences in the dendritic morphology hippocampal neurons are primarily due to differences in the number of non-primary dendrites. Male vs. female neuron-glia co-cultures were established from P0 hippocampi, transfected with MAP2B-GFP plasmid on day *in vitro* (DIV) 6 and fixed on DIV 9. Dendritic morphology was assessed by quantifying: (**a**) the number of primary dendrites; (**b**) the summed length of primary dendrites; (**c**) the mean length of primary dendrites; (**d**) the number of non-primary dendrites; (**e**) the summed length of non-primary dendrites; and (**f**) the mean length of non-primary dendrites. In the box plots, “+” indicates the mean; whiskers, the 10–90^th^ percentile (n = 76–101 neurons per sex from at least five independent dissections). Significant differences were determined using Student’s T-test for parametric data (**e**) and Mann-Whitney U test for nonparametric data (**a**,**b**,**c**,**d**,**f**). Asterisk indicates a significant difference between groups at p ≤ 0.05.

We further characterized dendritic architecture *in vitro* by quantifying the number and length of primary dendrites and non-primary dendrites, with the latter defined as those that branch off the primary dendrites (including secondary, tertiary etc.). The total number, sum length and mean length of primary dendrites did not differ significantly between male and female hippocampal neurons (Fig. 3a–c, p = 0.93, p = 0.62, p = 0.45 respectively). In contrast, the mean number of non-primary dendrites (Fig. 3d, p < 0.0001) and sum length of non-primary dendrites (Fig. 3e, p < 0.0001) are significantly greater in male vs. female hippocampal neurons, although the average non-primary dendritic length is not significantly different between sexes (Fig. 3f, p = 0.93).

**Figure 4:**
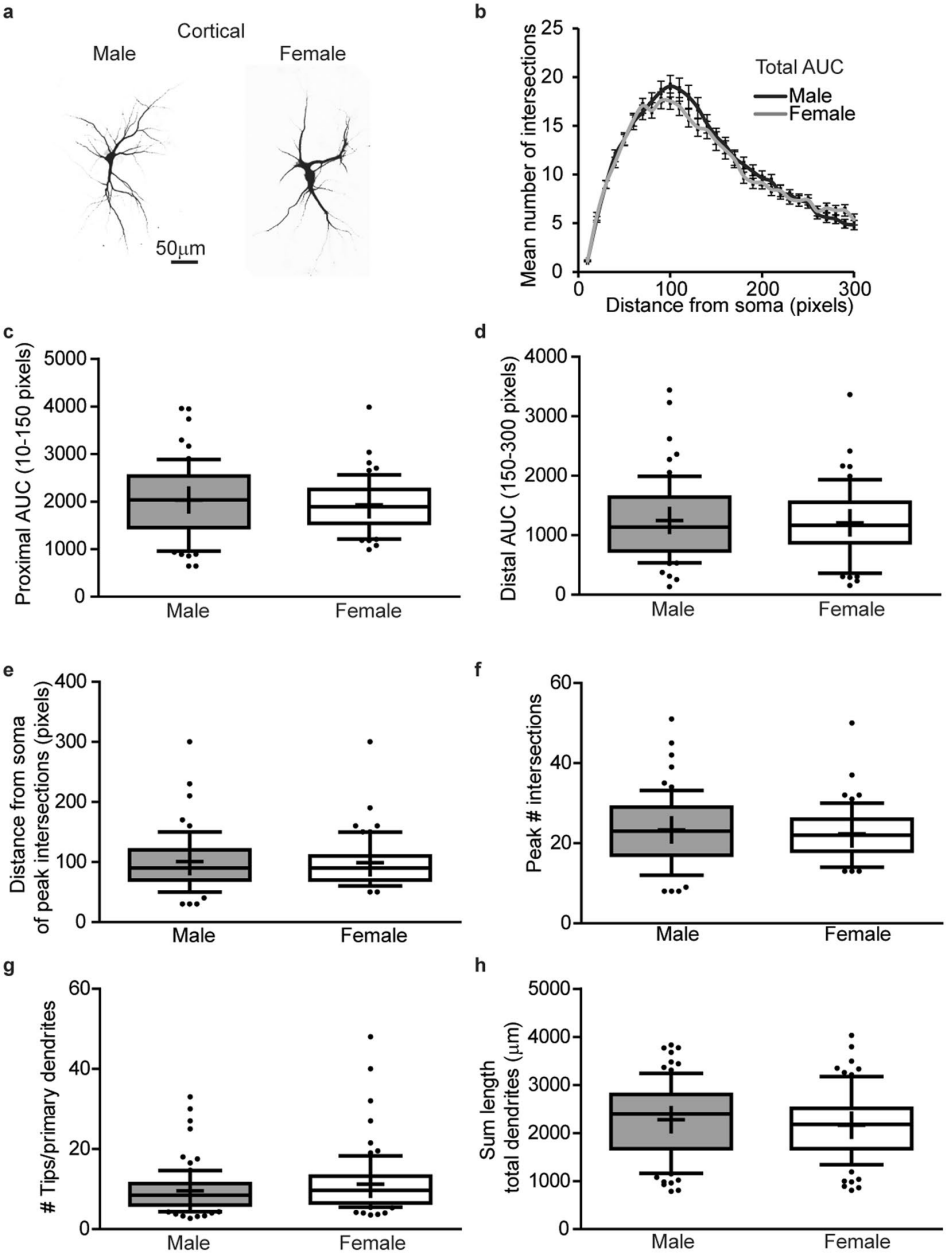
In cortical cultures, dendritic arbors are similar in male vs. female neurons. Sex-specific neuron-glia co-cultures were established from P0 mouse neocortex, transfected with MAP2B-GFP plasmid on day *in vitro* (DIV) 6 and fixed on DIV 9. Representative images (**a**) and Sholl plot (**b**) of DIV 9 GFP-positive male and female cortical neurons. Dendritic morphology was assessed by quantifying: (**b**) the total area under the Sholl curve (10–300 pixels from the soma); (**c**) the area under the proximal portion of the Sholl curve (10–150 pixels from the soma); (**d**) the area under the distal portion of the Sholl curve (150–300 pixels from the soma); (**e**) distance from the soma at which the peak number of dendritic intersections occurs; (**f**) the peak number of dendritic intersections; (**g**) the number of terminal dendritic tips per primary dendrite; and (**h**) the summed dendritic length. In box plots (**c-h**), “+” indicates the mean; whiskers, the 10–90^th^ percentile (n = 59–82 neurons per sex from at least five independent dissections). Significant differences were determined using Student’s T-test for parametric data (**h**) and Mann-Whitney U test for nonparametric data (**b**,**c**,**d**,**e**,**f**,**g**). No significant differences between groups were identified at p ≤ 0.05. AUC = area under the curve. Magnification; 0.65 microns per pixel.

**Figure 5:**
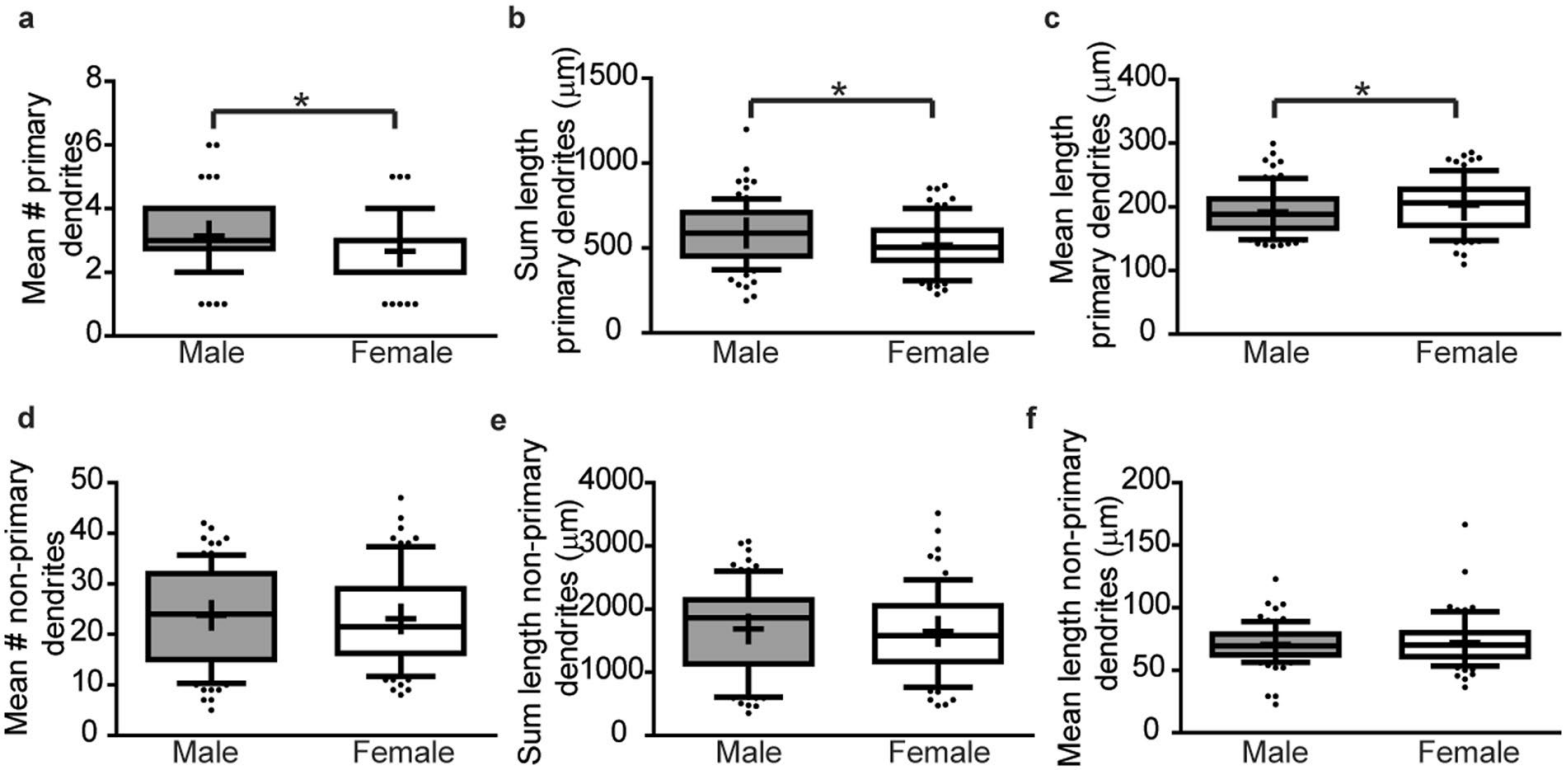
In cortical cultures, primary dendrite number is greater but average length less in male vs. female neurons. Sex-specific neuron-glia co-cultures were established from P0 mouse neocortices, transfected with MAP2B-GFP plasmid on day *in vitro* (DIV) 6 and fixed on DIV 9. Dendritic morphology was assessed by quantifying: (**a**) the number of primary dendrites; (**b**) the summed length of primary dendrites; (**c**) the mean length of primary dendrites, (**d**) the number of non-primary dendrites; (**e**) the summed length of non-primary dendrites; and (**f**) the mean length of non-primary dendrites. In box plots, “+” indicates the mean; whiskers, the 10–90^th^ percentile (n = 76–82 neurons per sex from at least five independent dissections). Significant differences were determined using Student’s T-test for parametric data (**b**), and Mann-Whitney U test for nonparametric data (**a**,**c**,**d**,**e**,**f**). Asterisk indicates a significant difference between groups at p ≤ 0.05.

*In vitro* sex differences in dendritic morphology are also observed in primary cortical neurons, although the differences are less pronounced than in hippocampal neurons (Fig. 4a). There are no significant differences in dendritic complexity as determined by area under the Sholl curve for the total dendritic arbor (Fig. 4b, p = 0.59) or for the proximal or distal halves of the dendritic arbor (Fig. 4c,d, p = 0.35, p = 0.89 respectively). Similarly, there are no significant differences in the distance from the soma of the peak number of dendritic intersections (Fig. 4e, p = 0.70) or in the peak number of intersections (Fig. 4f, p = 0.42). There is a trend for female neurons to have a greater number of terminal dendritic tips per primary dendrite compared to male neurons (Fig. 4g, p = 0.1); however, this is not statistically significant. There are also no significant sex differences in total dendritic length (Fig. 4h, p = 0.3). However, further analysis revealed subtle but significant sex differences in dendritic morphology of cortical neurons *in vitro*. Specifically, the mean length of primary dendrites is significantly greater in female than male cortical neurons (Fig. 5c, p = 0.0495), although female neurons have fewer primary dendrites (Fig. 5a, p = 0.0011) and the total length of primary dendrites is decreased in female compared to male neurons (Fig. 5b, p = 0.0085). There are no significant sex differences in the number (Fig. 5d, p = 0.5), sum length (Fig. 5e, p = 0.5) or mean length of non-primary dendrites (Fig. 5f, p = 1) in primary cortical cultures.

**Figure 6:**
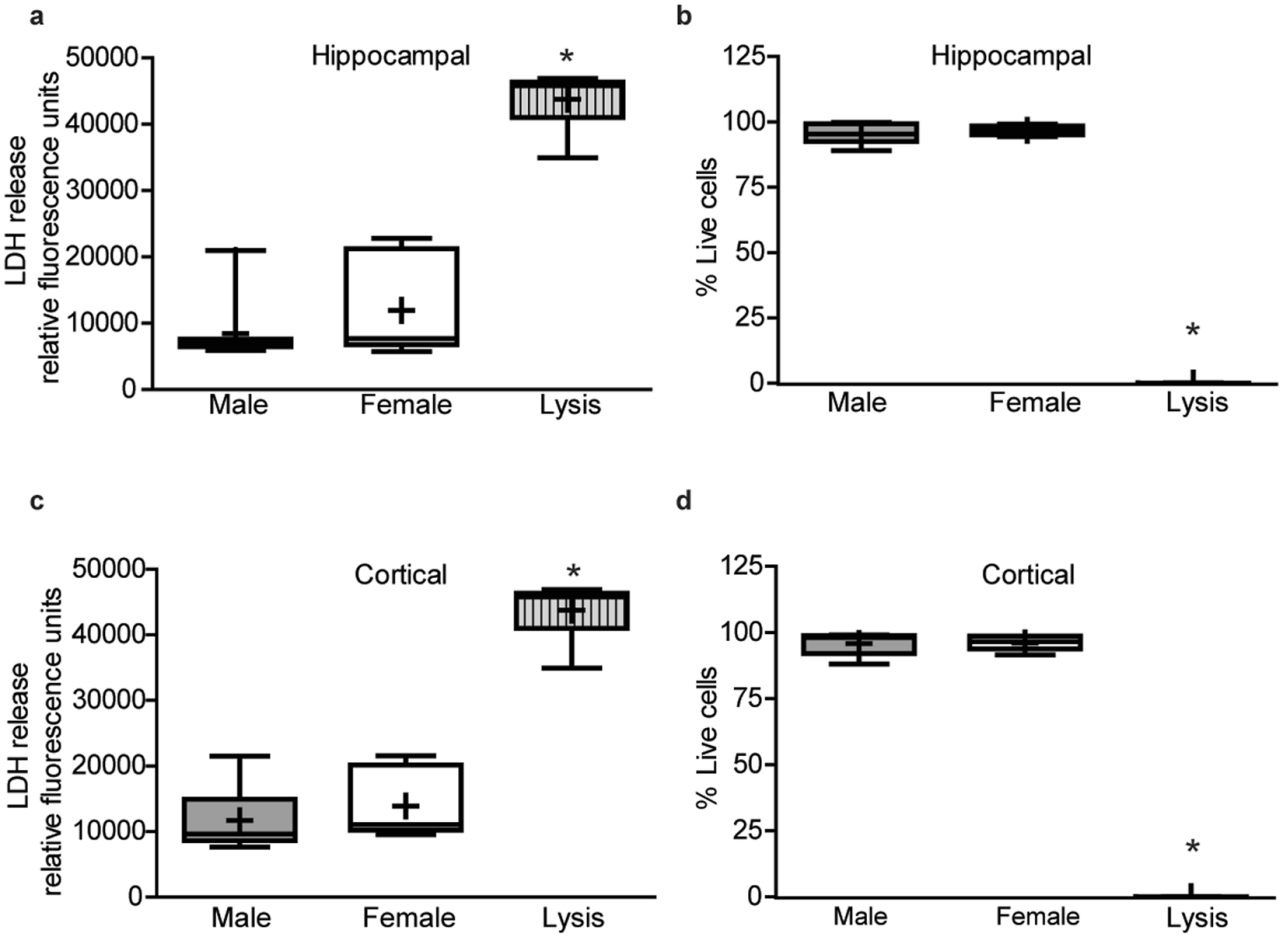
Cell viability does not differ by sex in hippocampal and cortical cultures. Sex-specific neuron-glia co-cultures were established from P0 mouse hippocampi (**a**,**b**) or neocortices (**c**,**d**). On DIV 9, cell viability was analyzed by measuring lactate dehydrogenase (LDH) release into the medium (**a**,**c**) or by quantifying the percentage of live cells in cultures stained with calcein AM (biomarker of live cells) and propidium iodide (biomarker of dead cells) using Metamorph image analysis software (**b,d**). As a positive control, a subset of cultures for each experimental condition were lysed with 0.2% Tritox X (Lysis). In box plots, “+” indicates the mean; whiskers, the 10–90^th^ percentile (n = 6–9 wells per sex from at least three independent dissections). Live dead cell analysis was conducted on imaged sites (9 fields/well) which contained greater than 250 total cells counted per field. Significant differences were determined using one-way analysis of variance (ANOVA) followed by Tukey’s *post hoc* analysis for parametric data (**b**) or Kruskal Wallis test followed by Dunn’s *post hoc* analysis for nonparametric data (**a**,**c**,**d**). Asterisk indicates a significant difference between groups at p ≤ 0.05.

To confirm that *in vitro* sex differences in dendritic morphology were not due to differences in cell viability of male vs. female cultures, a subset of cultures grown under the same conditions as cultures used for morphometric analyses were used to assess cell viability. Cell viability was determined by lactate dehydrogenase (LDH) release into the media, and by live dead analysis of cells stained with calcein AM and propidium iodide, which label live vs. dead cells, respectively. There were no significant differences in cell viability between male and female hippocampal (Fig. 6a,b, p = 0.25, p = 0.40 respectively) or cortical cultures (Fig. 6c,d, p = 0.38, p = 0.80 respectively) as determined by either method, and all experimental groups differed significantly from positive lysis controls (hippocampus: LDH p = 0.0011, live dead p = 0.0001; cortex: LDH p = 0.0005, live dead p = 0.0001).

### Estrogen signaling contributes to sex differences in dendritic complexity *in vitro*

Sex hormones and hormone receptors can modulate neural morphology and function^16, 54^, suggesting sex differences in dendritic morphology may be mediated by signaling through sex hormones. Testosterone and estrogen are produced by both sexes during the developmental periods at which we evaluated dendritic growth and both are critical for normal neurodevelopment^13, 55, 56^. Masculinization of the rodent male brain occurs in large part through the conversion of testosterone to estradiol by the enzyme aromatase which is found at high levels in the developing brain^13, 54–56^. Thus, we tested the hypothesis that estrogen mediates sex differences in dendritic morphology *in vitro*. As an initial test of this hypothesis, we quantified transcript levels of the androgen receptor (*Ar*), estrogen receptor alpha (*Esr1*), estrogen receptor beta (*Esr2*) and aromatase (*Cyp19a1*) in our model systems. All four transcripts were detected in the hippocampus and cortex of P28 mice and in primary cell cultures derived from these brain regions (Table 1). Therefore, we next evaluated a causal role for estrogen in sex differences in dendritic morphology in primary hippocampal cell cultures. This model was chosen because hippocampal neurons exhibited more robust sex differences in dendritic morphology than cortical neurons.

**Table 1:** Relative mRNA abundance.

*In vivo* postnatal day 28				
*Hippocampus*	*Ar*	*Esr1*	*Esr2*	*Aromatase*
Male	0.11 ± 0.02	0.0038 ± 0.0008	0.0027 ± 0.001	0.015 ± 0.006
Female	0.063 ± 0.01	0.0027 ± 0.0007	0.0011 ± 0.0004	0.0038 ± 0.001
***Cortex***				
Male	0.021 ± 0.004	0.0028 ± 0.0007	0.0022 ± 0.001	0.011 ± 0.005
Female	0.030 ± 0.003	0.012 ± 0.005	0.02 ± 0.01	0.12 ± 0.07
***Day in vitro 9***				
***Hippocampal***	***Ar***	***Esr1***	***Esr2***	***Aromatase***
Male	0.014 ± 0.002	0.0013 ± 0.0003	0.00028 ± 0.0002	0.00054 ± 0.0002
Female	0.017 ± 0.003	0.0019 ± 0.003	0.00046 ± 0.00008	0.00090 ± 0.0003
***Cortical***				
Male	0.014 ± 0.001	0.0015 ± 0.0002	0.0013 ± 0.0005	0.0013 ± 0.0004
Female	0.015 ± 0.002	0.0019 ± 0.0001	0.0013 ± 0.0002	0.0012 ± 0.0004

Results reported as mean ΔΔCt ± SEM. Abbreviations: *Ar*, Androgen receptor; *Esr1*, estrogen receptor alpha; *Esr2*, estrogen receptor beta.

**Figure 7:**
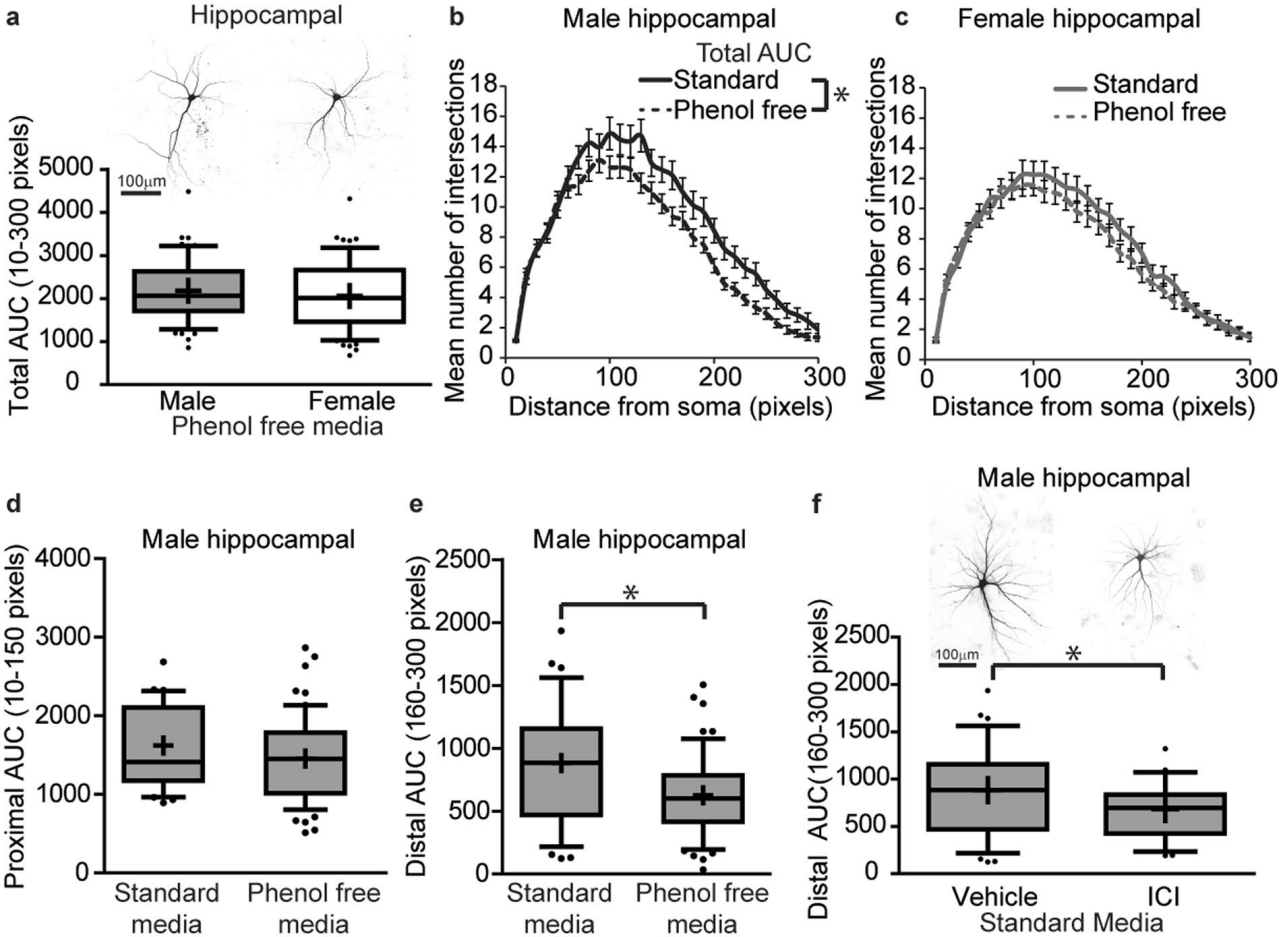
*In vitro* sex differences in dendritic morphology of hippocampal neurons is mediated by estrogen-dependent mechanisms. Sex-specific neuron-glia co-cultures were established from P0 mouse hippocampi, grown in standard culture medium containing phenol red or in phenol red-free medium, transfected with MAP2B-GFP plasmid on day *in vitro* (DIV) 6, treated with vehicle (0.05% DMSO) or estrogen receptor antagonist (ICI) on DIV 7 and fixed on DIV 9. As shown in representative images and box plots illustrating the total area under the curve (AUC) of Sholl plots of DIV 9 GFP-positive male and female hippocampal neurons (**a**), sex differences in dendritic morphology are not observed when neurons are cultured in medium without phenol red. Sholl plots of male (**b**) and female (**c**) hippocampal cultures grown in culture medium with or without phenol red; dendritic morphology was assessed by quantifying the total area under the Sholl curve. Dendritic morphology of male hippocampal neurons grown in culture media with or without phenol red was quantified by measuring: (**d**) the proximal (10–150 pixels from the soma) and (**e**) distal (160–300 pixels from soma) area under the curve (AUC). Representative images and Sholl analyses of distal AUC of DIV 9 GFP-positive male hippocampal neurons grown in standard culture media and treated with either vehicle or the estrogen receptor antagonist ICI (1 μM) (**f**). In box plots (a, d-f), “+” indicates the mean; whiskers, the 10–90^th^ percentile (n = 23–60 neurons per sex per group from at least three independent dissections). Significant differences were determined using Student’s T-test for parametric data (**a**,**b**,**c**,**e**,**f**) and Mann-Whitney U test for nonparametric data (**d**). Asterisk indicates a significant difference between groups at p ≤ 0.05. AUC = area under the curve. Magnification; 0.65 microns per pixel.

To modulate estrogen signaling in hippocampal cell cultures, we cultured male and female hippocampal neurons in phenol red-free medium. Most standard culture media, including the medium used in initial studies of *in vitro* sex differences in this study, contain phenol red as an indicator of pH. Phenol red can act as an estrogen^57^, and in neuronal cell cultures has been shown to mimic effects of estrogen on excitability^58^. In contrast to hippocampal neurons grown in standard phenol red-containing culture medium that exhibit marked sex differences in dendritic morphology (Fig. 2), there are no significant differences in the dendritic morphology of male vs. female neurons dissociated from the same tissue preparation but cultured in phenol red-free culture medium (Fig. 7a, p = 0.30). This was due to an effect of phenol red on the dendritic arborization of male hippocampal neurons as evidenced by a significant decrease in dendritic complexity, as determined by quantification of the total area under the Sholl curve, in male neurons cultured in phenol red-free media compared to standard media containing phenol red, (Fig. 7b p = 0.007). In contrast, the dendritic complexity of female hippocampal neurons was not altered significantly by removal of phenol red from the culture medium (Fig. 7c p = 0.30). Further characterization of the decreased dendritic complexity observed in male neurons cultured in medium lacking phenol red relative to their counterparts cultured in the presence of phenol red, identified no significant difference in the area under the Sholl curve for the proximal half of the dendritic arbor (intersections occurring from 10–150 pixels from soma) (Fig. 7d, p = 0.1), but indicated a significant decrease in the area under the Sholl curve for the distal half of the dendritic arbor (intersections occurring from 160–300 pixels from the soma) (Fig. 7e, p = 0.005). Thus, culturing in phenol red-free media decreased dendritic complexity of male neurons to the level of dendritic complexity observed in female neurons in large part by decreasing dendritic complexity in the distal half of the arbor. To confirm that the effect of phenol red on the dendritic morphology of male hippocampal neurons is mediated by its estrogenic activity, we determined whether pharmacological antagonism of estrogen receptors phenocopied the effect of removing phenol red from the culture medium. Male hippocampal neurons cultured in the presence of the estrogen receptor antagonist ICI 182,780 (ICI) were less complex in the distal portion of the dendritic arbor (intersections occurring from 160–300 pixels from soma) compared to vehicle controls (Fig. 7f, p = 0.040).

## Discussion

This study describes novel data demonstrating sex differences in the dendritic morphology of pyramidal neurons of the juvenile mouse CA1 hippocampus and adjacent somatosensory cortex. Sex-dependent effects are region-specific with dendritic arbors of male neurons being more complex than those of female neurons in the hippocampus, while the opposite is true in the cortex. Analyses of primary cell cultures revealed that sex differences are also observed *in vitro*, with pronounced differences observed in hippocampal neurons and, more subtle differences detected in cortical neurons. In hippocampal cultures, male neurons exhibit greater complexity than female neurons, driven largely by differences in non-primary dendrite number across both proximal and distal portions of the dendrite. In cortical cultures, more subtle sex differences are observed with female cortical neurons exhibiting fewer but longer primary dendrites relative to male cortical neurons.

In the developing mouse hippocampus, robust sex differences in dendritogenesis were observed *in vivo* and *in vitro*, and in both models, male neurons elaborate more complex dendritic arbors than female neurons. While these models are not directly comparable, the fact that similar differences were observed at two very different stages of development and under different environmental conditions, suggests that some sex differences in dendrite biology may be determined early in life. Sex differences observed at P28 may also reflect known sex differences in the developmental trajectory of the hippocampus, i.e., the rate of neurogenesis in the hippocampus during the first week after birth is greater in males compared to females^54, 56^. These findings are consistent with previous studies of the adult rat hippocampus, which illustrated that the dendritic field volume and soma size of dentate gyrus granule cells and CA1 pyramidal cells are greater in males vs. females^24, 28^. Similarly, our observations of proximal-distal differences in dendritic complexity are also consistent with previous studies. In adult rat CA3 hippocampal neurons, sex differences in dendritic complexity are opposite in proximal (female >male) and distal (male >female) portions of the arbor^43^. This is important to consider since it has previously been shown that *in utero* and lactational exposure to environmental neurotoxicants, specifically, polychlorinated biphenyls (PCBs), decreases the dendritic complexity of basilar CA1 pyramidal hippocampal neurons in juvenile male rats (P22), but only in distal portions of the arbor^59^. Interestingly, when examined in adult male rats (P60), *in utero* and lactational exposure to PCBs causes a significant increase in complexity, but again only in the distal portion of the arbor^59^. This study only examined male rats, which raises the intriguing question of whether underlying sex-dependent differences in proximal-distal dendritic complexity contribute to sexually dimorphic effects of neurotoxicants such as PCBs. These results also underscore the importance of not relying on a single aspect of dendritic morphology when studying sex-dependent effects of environmental neurotoxicants since effects may vary depending on where in the dendritic arbor data are collected.

A notable finding in this study was that sex differences in dendritic morphology varied regionally. In the hippocampus, male neurons exhibited significantly more complex dendritic arbors than female neurons in both *in vivo* and *in vitro* models. In contrast to hippocampal neurons, we observed more subtle sex differences in cortical neurons. In the P28 brain, sex differences consisted primarily of female cortical neurons exhibiting greater dendritic complexity than male cortical neurons. In cultured cortical neurons, sex differences were limited, with female neurons exhibiting longer but fewer primary dendrites than male neurons. The difference in age and heterogeneity of neurons present in the somatosensory cortex at the time of analysis likely explain the differences in the extent and type of sex differences observed in cortical neurons *in vivo* and *in vitro*. The somatosensory cortex consists of a heterogeneous population of neurons organized in a multi-layered structure with neurons at different stages of maturation within the layers^60^. For *in vivo* analyses, we focused on P28 pyramidal neurons within Layer 4–5. For *in vitro* studies, the entire P0 cortex is dissociated to establish primary cultures thus making it technically impossible to only focus on a single layer. Moreover, not all layers are represented in the primary cultures since neurons of the upper layers are still migrating in at P0^60^. Thus it is not surprising that trends in sex differences for pyramidal neurons of the somatosensory cortex in each model were more variable and the correlation between the types of sex differences observed *in vivo* vs. *in vitro* were not as pronounced as in the case of the hippocampus. *In vivo*, differences in the maturation of somatosensory cortical neurons may explain the observed sex difference of females having greater dendritic complexity than males, which was the opposite trend observed in P28 CA1 pyramidal hippocampal neurons. The prefrontal cortex is one of the later brain regions to mature, with dendritic maturation and pruning occurring in this brain region primarily during adolescence^12, 61^. In rats, dendritic complexity in the cortex increases in puberty from P20 to P35^61^; however, rates of growth differ between the sexes. Ramification of prefrontal cortex neurons occurs earlier in females than males and also differs in location, with females showing growth across the dendritic arbor while males exhibit the most growth in the middle portion of the dendritic arbor^61^. This may explain why we saw greater complexity in female vs. male cortical neurons at P28 while others have reported the opposite in studies of adult rat cortex, in which male neurons exhibit increased dendritic length, complexity and spine density compared to female neurons^12, 41, 42, 62^.

Mechanistic studies of sex differences in primary neuron-glia co-cultures indicate that these differences are not hardwired, but rather they are malleable, as evidenced by the impact of changing culture conditions on male dendritic arbors. Specifically, sex differences in dendritic arborization in cultured hippocampal neurons are mitigated by removing the estrogenic compound phenol red from the culture medium, or by pharmacological antagonism of estrogen receptors. These findings are consistent with previous studies showing that estrogen augments neurite outgrowth from explants of the hypothalamus or preoptic area^63^. These data suggest that the mechanisms driving sex differences in dendritic arborization, at least in hippocampal pyramidal neurons involve estrogen, an observation consistent with a significant body of literature indicating that the growth and maturation of neurons is hormone-dependent, with estrogen playing a major role^13^, as well as numerous studies demonstrating that estrogens increase dendritic spine number and density in adult male rat neurons of the dentate gyrus, and that this effect can be completely blocked by addition of ER antagonist, ICI^64^. These effects are especially evident in the hippocampus where adult male rat estradiol levels are higher in the hippocampus than in the systemic circulation^65^.

The findings from our *in vitro* experiments have a practical implication in that they show that culture conditions commonly used in neurodevelopmental studies can significantly influence dendritic morphology. Therefore, interpretation of data generated using single sex or mixed sex cultures should include a consideration of the influence of media components or other endocrine signaling components in the culture system. It should be noted that phenol red-free media is not meant to be interpreted here as a ‘hormone free’ culture condition. Primary cultures of rat hippocampal neurons are capable of producing hormones, including estrogen, *in vitro* ^66^, and common growth supplements added to growth media often contain corticosterone and progesterone. It would be valuable to determine levels of steroid hormones present in standard growth media, phenol free growth media, the cells themselves or carried over from brain tissue during dissection.

Dendritic development can be broadly separated into two phases: (1) primary dendritogenesis which includes initiation of dendritic growth and extension of primary dendritic shafts; and (2) dendritic maturation which encompasses dendrite branching and elongation, spine formation and pruning^67^. We did not observe differences between the sexes with respect to the number of primary dendrites in pyramidal neurons from the CA1 hippocampus or adjacent pyramidal somatosensory cortex in P28 brain sections or in pyramidal neurons in hippocampal cell cultures. This suggests that the sex differences in dendritic arborization observed in these models likely occur during the later stage of dendrite maturation, which is consistent with literature suggesting that primary dendritogenesis is largely determined by intrinsic factors whereas dendritic maturation is strongly influenced by extrinsic factors. What is not clear from these studies is whether the sex differences we observed reflect differences between the sexes in dendritic branching and elongation or in dendritic pruning. During the dynamic process of establishing neural connectivity, dendrites extend and eliminate branches at a high rate^68–70^. Pruning occurs in both sexes, however, evidence suggests that the rates may vary. For example, dendritic pruning occurs in the medial prefrontal cortex of female, but not male, rats from P35 to P90^71^. Dendritic branch survival also depends on the strength of the synaptic connections established. Branches are preferentially formed in regions of active synapse formation and the rates of addition and pruning are affected by neural electrical activity^62, 69, 70, 72^. Whether sex differences in pruning or neural electrical activity contribute to the brain region specific sex differences in primary and non-primary dendrite arbor morphology observed remain to be determined.

Our findings capture a snapshot of the sex differences observed in mouse CA1 hippocampal and adjacent somatosensory cortical neurons during early postnatal development. Whether these differences persist into later stages of development was not examined here, however, evidence from rodent and human data suggest that at least some of these differences persist and have functional consequences. Adult rat studies illustrate that CA1 pyramidal cell field volume and soma size are greater in male vs. female neurons^24, 28^. The dentate gyrus granule cell layer of the adult rat hippocampus is also larger in male compared to female animals, and is correlated with performance in behavioral tasks of learning and memory, such as the radial arm maze and Morris Water Maze, where it is recognized that male rats perform better than female rats^27, 73, 74^. Human studies also indicate that hippocampal volumes increase in both sexes during adolescence but eventually diverge, increasing in males and slightly decreasing in females^12, 32, 35^. Future studies to address the growth trajectories of these endpoints in mice would greatly add to our knowledge of sex differences in dendrite morphology of the developing brain and help in guiding studies that employ these models to investigate NDDs.

The question in the field remains as to what drives the sex bias in the onset, prevalence and severity for many NDDs. Given the central role of neuronal connectivity in many of these NDDs^39, 40^, our data suggest that inherent sex differences in dendritic morphology, which is a critical determinant of neuronal connectivity^29–31^ may in part contribute to the sex bias observed in NDDs. In line with our findings, human studies examining functional connectivity in the brain indicate that not only do typically developing males and females have different patterns of connectivity (male >female) but also that male and female ASD patients differ in their patterns of connectivity^75^. Thus, a pattern of hyperconnectivity is observed in ASD female vs. typical female, whereas a pattern of hypoconnectivity is observed in ASD male vs typical male, suggesting that ASD females shift towards a male pattern while ASD males shift towards a female pattern^75^. Disruption in neural masculinization/feminization patterns in ASD has also been reported for neuroanatomy^76^, task based brain activity^77^ and cognitive profiles^78^. In summary, our findings establish that there are significant sex differences in the dendritic morphology of typically developing CA1 pyramidal hippocampal and adjacent pyramidal somatosensory cortical neurons in juvenile mice and in primary culture. These results highlight the importance of examining sexually dimorphic effects of genetic mutations and environmental stressors on dendritic morphology in models of neurodevelopment, particularly in the context of NDDs where sex-specific patterns of connectivity likely impact NDD risk and/or severity.

## Methods

### Animals

All procedures involving animals were conducted in accordance with the NIH Guide for the Care and Use of Laboratory Animals and were approved by the University of California Davis Animal Care and Use Committee (IACUC protocol numbers 18853 and 18682). C57BL/6 J wild type mice were purchased from Jackson Labs (Sacramento, CA) and housed in clear plastic cages containing corn cob bedding. Mice were maintained on a 12 h reverse light and dark cycle at 22 ± 2 °C. Feed (Diet 5058, LabDiet, Saint Louis, MO) and water were available *ad libitum*. Female mice were paired overnight with males to obtain timed-pregnant dams.

### Golgi Staining

Golgi staining was performed using the FD Rapid GolgiStain kit (FD NeuroTechnologies Inc.) according to the manufacturer’s instructions with the following modifications. After immersion in Solution C, brains were transferred to 10% sucrose in phosphate-buffered saline (PBS) for 3–4 h at 4 °C, and then stored in 30% sucrose in PBS for 3–4 days at 4 °C until sectioned. Prior to sectioning, brains were transferred to 70% ethanol in distilled water for 4 h at 4 °C. Brains were cut into 100-µm thick coronal sections using a vibratome (VT-1000, Leica, Solms, Germany). Sections were mounted on gelatin-subbed slides and stained according to the manufacturer’s instructions. Brightfield image stacks of CA1 hippocampal pyramidal neurons and pyramidal somatosensory cortical neurons adjacent to the CA1 hippocampus were acquired with an IX-81 inverted microscope (Olympus, Shinjuku, Japan) using MetaMorph Image Analysis Software (version 7.1, Molecular Devices, Sunnyvale, CA) by an individual blinded to sex. Criteria for selection of neurons for Golgi analyses have been described previously^59^. Additionally, criteria for the CA1 hippocampus were that the cell body was located in the middle third of the thickness of the section and in the somatosensory cortex cell body was located in Layer 4–5. Pyramidal neurons were selected based on their cytoarchitecture, including a clearly identifiable axon and apical dendrite. Basilar dendritic arbors of selected neurons were traced using NeuroLucida (version 11, MBF Bioscience, Williston, VT) and arbor complexity was quantified by branch structure analysis and Sholl analysis using NeuroLucida Explorer (version 11, MBF Bioscience)^59^.

### Cell Culture

Primary neuron-glia co-cultures dissociated from the neocortex and hippocampus of P0 mouse pups were prepared as previously described^52^. Sex determination was confirmed by examination of the sexually dimorphic lower urinary tract to confirm the presence of testes or ovaries^79–81^. Cortical and hippocampal neurons were plated on German glass coverslips (Bellco Glass Inc., Vineland, NJ) precoated with poly-L-lysine (Sigma-Aldrich) at a density of 83,000 cells/cm^2^ and maintained at 37 °C in NeuralQ Basal Medium (GSM-9420; MTI-GlobalStem, Gaithersburg, MD) or NeuralQ Basal Medium minus phenol red (GSM-9421; MTI-GlobalStem) supplemented with 2% GS21 (GSM-3100; MTI-GlobalStem) and 1% GlutaMAX (ThermoScientific, Waltham, MA) under 5% CO_2_. On DIV 4, half the volume of culture medium was replenished and cytosine β-D-arabinofuranoside hydrochloride (Ara-C) (Sigma-Aldrich, St. Louis, MO) added at a final concentration of 2.5 μM to curb glial cell proliferation. On DIV 6, cells were transfected with plasmid encoding microtubule-associated-protein-2B (MAP2B) fused to either enhanced green fluorescent protein (EGFP) or red fusion (MAP2B-FusRed) construct (provided by Dr. Gary Wayman, Washington State University, Pullman, WA)^53, 82^ using Lipofectamine-2000 (Invitrogen, Carlsbad, CA) according to the manufacturer’s protocol. DIV 7 cultures were treated for 48 h with vehicle (DMSO; 0.05%) to mimic common dosing paradigms used in assessing dendritic growth in response to environmental contaminants, drugs and other molecular targets^52, 53^. In a subset of studies, DIV 7 cultures were treated for 48 h with either vehicle or the estrogen receptor antagonist ICI 182, 780 at 1 μM (ab120131; Abcam, Cambridge, MA). At DIV 9, cultures were fixed with 4% paraformaldehyde and mounted to glass slides using ProLong Gold antifade reagent with DAPI (ThermoScientific). Slides were imaged using ImageExpress Micro XL high content imaging system (Molecular Devices, Sunnyvale, CA) to obtain automated non-biased acquisition of EGFP or FusRed positive neurons from each group^83^. The dendritic complexity of individual neurons was quantified using ImageJ software^84^ with Sholl analysis plug-in v3.4.2 (http://fiji.sc/Sholl_Analysis)^85^ and with NeuronJ plug-in^86^ to quantify dendritic length, magnification used for image analysis was 0.65 microns per pixel.

### Cell Viability

Cell viability was tested in DIV 9 cultures. Lactate dehydrogenase (LDH) release was measured using the CytoTox-ONE™ Homogenous Membrane Integrity Assay (Promega, Madison, WI, USA) per the manufacturer’s instructions. Cell viability was also assessed in separate cultures co-stained with calcein-AM (0.25 μM, ThermoFisher Scientific) and propidium iodide (1.25 μM, Sigma-Aldrich) to identify live vs. dead cells, respectively. Lysis control wells were incubated with 0.2% Triton X for 2 min. After a 30 min at 37 °C incubation in calcein AM and propidium iodide, cultures were washed in phosphate-buffered saline (PBS) and imaged using the ImageXpress Micro XL high content imaging system (Molecular Devices). The number of calcein-AM and propidium iodide-stained cells was quantified using MetaXpress 5.0 software (Molecular Devices), and the % live cells determined as the number of calcein-AM stained cells divided by the sum of the number of calcein-AM and propidium iodide-stained cells per field.

### Quantitative Real Time PCR (QPCR)

RNA was purified from tissues using the Qiagen RNeasy kit (Qiagen, Gaithersburg, MD) and from DIV 9 cultures using TRIzol reagent (ThermoFisher Scientific) according to the manufacturer’s instructions. RNA was reverse transcribed using the SuperScript VILO cDNA synthesis kit (ThermoFisher Scientific) according to manufacturer’s instructions. Real time PCR was performed in 12.25 μl reactions containing 6 μl POWER SYBR Green PCR Master Mix (ThermoFisher Scientific), 0.2 μM PCR primers (0.25 μl of 10 μM stock), 5 μl of cDNA and 1 μl of water and amplified using an Applied Biosystems 7900HT Fast thermocycler at the Real-Time PCR Research and Diagnostics Core Facility (UC Davis). PCR conditions were 50 °C for 2 min, 95 °C for 10 min, then 40 cycles of 95 °C for 15 sec, 60 °C for 30 sec and 72 °C for 30 sec. QPCR was conducted on n = 6–8 tissues/wells per sex per brain region from at least three mice from independent litters or three dissections from independent litters using gene specific primers for androgen receptor (*Ar*) F 5′-GATGGTATTTGCCATGGGTTG-3′ and R 5′-GGCTGTACATCCGAGACTTGTG-3′; estrogen receptor alpha (*Esr1*) F 5′-GCTGTGCACCCTGGAAATAAC-3′ and R 5′-CTCGACGACCAATGACCTCTC-3′; estrogen receptor beta (*Esr2*) F 5′-CATCCAGAAGATGTGGTGCTG-3′ and R 5′-GCTGCCTGTGGAAGACAAAG-3′; aromatase (Cyp19a1) F 5′- TGAGACGATTCCAGGTGAAGAC-3′ and R 5′- TGTCCTCATTTGGGTGCAAG-3′; peptidylprolyl isomerase a (*Ppia*) F 5′-TCTCTCCGTAGATGGACCTG-3′ and R 5′-ATCACGGCCGATGACGAGCC-3′; hypoxanthine guanine phosphoribosyl transferase (*Hprt*) F 5′-GGAGAGCGTTGGGCTTACCT-3′ and R 5′-CGGCAAAAAGCGGTCTGAGG-3′; and phosphoglycerate kinase 1 (*Pgk1*) F 5′- CCCATGCCTGACAAGTACTCC-3′ and R 5′-ACAGGCATTCTCGACTTCTGG-3′. Relative mRNA abundance was determined by the ΔΔCt method as described previously^87^ and normalized to the geometric mean of references genes *Ppia*, *Hprt* and *Pgk1* abundance.

### Statistics

Golgi stained neurons from P28 brain sections were analyzed as described previously^45^. Briefly, we used the SAS® software procedure MIXED for all mixed effects models to account for clustering of neurons within animal. We used the variance components option to specify the covariance structure for all models except for the Sholl profile with multi-level nesting where we used an autoregressive (AM(1)) covariance structure. All analyses were conducted using SAS® software version 9.4 of the SAS System for Windows® (SAS Institute Inc, Cary, NC). For multi-level data, if an outcome variable did not appear to be normal based on histograms, summary statistics and residual plots, or if unequal variances were observed, the appropriate transformation was used to achieve approximate normality and Satterthwaite degrees of freedom were used in the mixed model to account for unequal variance. For all other endpoints, data were assessed for normality and homogeneity of variance using the Shapiro-Wilks test and F test respectively using GraphPad Prism v 6.07 (San Diego, CA). Differences between groups were assessed using Student’s T-test or Student’s T-Test with Welch’s correction for parametric data and by the Mann Whitney U test for nonparametric data. Cell viability assays were analyzed using a one way analysis of variance (ANOVA) followed by Tukey’s multiple comparison test for parametric data or Kruskal-Wallis test with Dunn’s multiple comparison test for nonparametric data. Area under the curve for the number of dendritic intersections, distance from soma of peak intersection (peak X), and maximum dendritic intersections (peak Y) values were calculated for Sholl profiles using built in area under the curve analysis in GraphPad Prism Software. Data are reported as mean ± SEM for Sholl plots and box and whisker plots indicate the mean as a (+) and whiskers are 10–90^th^ percentile, p values ≤0.05 were considered significant.

### Data Availability

The datasets generated during and/or analyzed during the current study are available from the corresponding author on reasonable request.
